# Identification of the Neogenin-Binding Site on the Repulsive Guidance Molecule A

**DOI:** 10.1371/journal.pone.0032791

**Published:** 2012-03-01

**Authors:** Takahide Itokazu, Yuki Fujita, Ryosuke Takahashi, Toshihide Yamashita

**Affiliations:** 1 Department of Molecular Neuroscience, Graduate School of Medicine, Osaka University, Suita, Osaka, Japan; 2 Core Research for Evolutional Science and Technology, Japan Science and Technology Agency, Chiyoda-ku, Tokyo, Japan; 3 Department of Neurology, Graduate School of Medicine, Kyoto University, Sakyo-ku, Kyoto, Japan; Kyushu University, Japan

## Abstract

Repulsive guidance molecule (RGM) is a membrane-bound protein that was originally identified as an axon guidance molecule in the chick retinotectal system. RGMa, one of the 3 isoforms found in mammals, is involved in laminar patterning, cephalic neural tube closure, axon guidance, and inhibition of axonal regeneration. In addition to its roles in the nervous system, RGMa plays a role in enhancing helper T-cell activation. Binding of RGM to its receptor, neogenin, is considered necessary to transduce these signals; however, information on the binding of RGM to neogenin is limited. Using co-immunoprecipitation studies, we have identified that the RGMa region required for binding to neogenin contains amino acids (aa) 259–295. Synthesized peptide consisting of aa 284–293 directly binds to the extracellular domain (ECD) of recombinant neogenin, and addition of this peptide inhibits RGMa-induced growth cone collapse in mouse cortical neurons. Thus, we propose that this peptide is a promising lead in finding reagents capable of inhibiting RGMa signaling.

## Introduction

Repulsive guidance molecule (RGM) is a cell membrane-associated glycosylphosphatidylinositol (GPI)-anchored glycoprotein that was originally identified as an axon repellent in the chick retinotectal system [1], [2]. This protein contains an N-terminal signal peptide, an Arg-Gly-Asp (RGD) site, a partial von Willebrand type-D (vWD) domain, and a hydrophobic domain of unknown function [3]. It also has a putative proteolytic site, and the cleaved carboxy-terminal mature protein is a 33-kDa fragment [4], [5]. In vertebrates, at least 3 homologues of RGM, RGMa, RGMb (DRAGON), and RGMc (hemojuvelin HJV, HFE2), have been identified [6], [7]. RGMa is the most closely related to chick RGM with 80% homology. Although overexpression or downregulation of RGM in chick tectum results in pathfinding and mapping errors, RGMa mutant mice show normal retinal axon projection patterns in the superior colliculus [6]. In contrast, another study showed that RGMa functions as an axon guidance molecule in the developing mouse hippocampus [8]. In addition, 50% of the homozygous mutant mice show defects in cephalic neural tube closure [6]. Moreover, both *in vitro* and *in vivo* studies have shown that RGMa inhibits axon growth [9]. RGMa expression is induced in adult rats with a spinal cord injury (SCI) around the lesion site, while loss of function by local treatment with a neutralizing antibody to RGMa significantly promotes axon regeneration after SCI.

Neogenin, the receptor for all of the RGM homologues, is a single membrane-embedded protein originally isolated from chick cerebellum as a homolog of deleted in colorectal cancer, a receptor for the axon guidance cue netrin-1 [10]. Neogenin contains 4 immunoglobulin-(IgG) like and 6 fibronectin type III-(FN III)-like domains in its extracellular region. Both netrin-1 and RGM bind to the FN III-like domain of neogenin, but the binding affinity of RGM for neogenin is much higher than that of netrin-1 for neogenin [10], [11]. Unfortunately, information on the binding site on RGM for neogenin has been lacking.

In this study, we identified that the region of RGMa containing aa 259–295 critically regulates the interaction between RGMa and neogenin. Results from enzyme-linked immunosorbent assays (ELISAs) revealed that the RGMa aa 284–293 directly bind to neogenin's extracellular domain. Furthermore, this peptide attenuated RGMa-induced growth cone collapse in mouse cortical neurons.

## Results

### C-terminus of RGMa binds to neogenin

**Figure 1:**
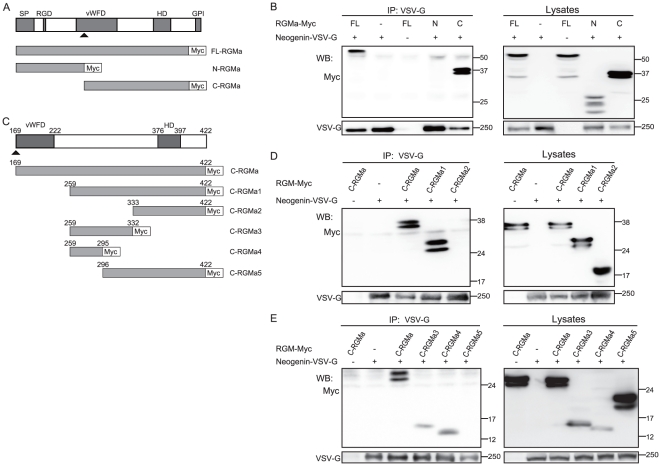
The RGMa domain required for binding to neogenin is within aa 259–295. (A and C) Schematic representation of RGMa and its deletion mutants are shown with their domain structures. Arrowhead shows potential cleavage site. SP: signal peptide, vWD: von Willebrand factor type-D domain, HD: hydrophobic domain, GPI: GPI-anchor. (B, D, and E) Co-immunoprecipitation of full-length neogenin-VSV-G with the deletion mutants of RGMa-Myc. HEK293T cells were transiently transfected with the indicated plasmids. Cell lysates were immunoprecipitated with the anti-VSV-G antibody. The immunoprecipitates (IP) and cell lysates (Lysates) were analyzed by western blotting with anti-Myc and anti-VSV-G antibodies.

First, we sought to determine whether the binding sites for neogenin are located in the amino- (N-) or carboxyl- (C-) terminus of RGMa. Deletion constructs were made consisting of Myc-tagged human full-length RGMa (FL-RGMa-Myc), N-terminal region of RGMa (N-RGMa-Myc), and C-terminal region of RGMa without a GPI anchor domain (C-RGMa-Myc) (Fig. 1A). HEK293T cells were transiently co-transfected with a control or VSV-G-tagged full-length neogenin constructs together with each of the RGMa constructs, and the cell lysates were immunoprecipitated with an anti-VSV-G antibody. Expression of FL-RGMa, N-RGMa, and C-RGMa was observed at approximately 55, 27, and 37 kDa, respectively, in the whole cell lysates (Fig. 1B, right panel), although multiple bands could be seen in each lane, as previously reported [2], [9]. These multiple bands may be due to the posttranslational modifications in the expressed proteins, because RGMa is considered to have three asparagine-linked glycosylation sites (two in the N-terminus and one in the C-terminus) [12]. FL-RGMa-Myc and C-RGMa-Myc co-immunoprecipitated with VSVG-Neogenin (Fig. 1B); however, N-RGMa did not. These results show that the C-terminus, but not the N-terminus of RGMa binds to neogenin.

### Neogenin-binding site resides between the vWD and the hydrophobic domain of RGMa

To more precisely determine the neogenin-binding site of RGMa, expression vectors encoding various fragments of Myc-tagged C-RGMa were generated (Fig. 1C), and their ability to bind to neogenin was assessed using co-immunoprecipitation as described above. Our results show that deletion of aa 169–258, including the vWD domain (C-RGM1), did not affect binding (Fig. 1D), while deletion of aa 169–332 (C-RGMa2) did, suggesting that the neogenin-binding site is localized within aa 259–332. To further pinpoint the neogenin-binding site of RGMa, shorter fragments were designed. Although some of the fragments were hardly expressed probably due to their shorter lengths, expression of 3 fragments—C-RGMa3, C-RGMa4, and C-RGMa5 (for sequences refer to Fig. 1C)—was detectable (Fig. 1E). Despite their limited expression, bands for C-RGMa3 and C-RGMa4 were clearly detected in the IP lanes (Fig. 1E), whereas C-RGMa5 did not co-immunoprecipitate with VSVG-Neogenin. Taken together, these results show that RGMa fragments containing aa 259–295 interact with neogenin, while fragments lacking this domain do not. Our immunoprecipitation experiments demonstrate that the RGMa domain required for binding to neogenin is within aa 259–295.

### Determination of the core binding site by ELISA

**Figure 2:**
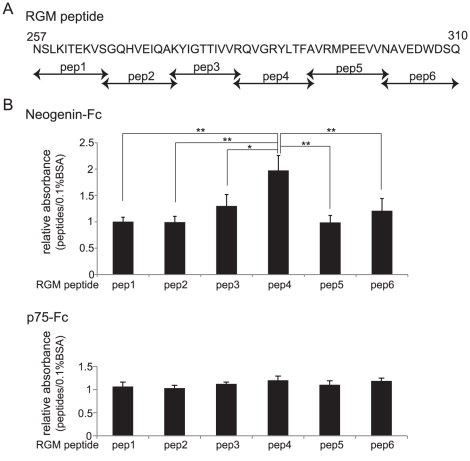
RGMa aa 284–293 are critical for the interaction with neogenin. (A) Sequence alignment of the synthesized RGMa peptide. Peptides 1–6 were designed within aa 257–310, which is the region required for the interaction between RGMa and neogenin as shown in Fig. 1. (B) RGMa peptide 4 (aa 284–293) directly binds to neogenin's ECD. Relative binding of each RGMa peptide to ELISA plates coated with neogenin-ECD-Fc is shown. p75NTR-Fc was used as a control. Results (%) are shown as the mean ± SEM of 3 independent experiments (**P<0.01, *P<0.05).

Direct binding of different RGMa peptide fragments to neogenin was assessed by ELISA. Six peptides (each 10-aa long) derived from aa 257–310 of RGMa (Fig. 2A) were assessed for their ability to bind to neogenin. Each RGMa peptide was coated on the ELISA plate, and then recombinant Fc-fused neogenin ECD or neurotrophin receptor p75 (p75NTR) ECD (negative control) was added. The results in Fig. 2B show that peptide 4 (aa 284–293) had a significantly higher binding affinity for neogenin compared with other RGM peptides. Peptide 3 showed some tendency to bind to neogenin, but this was not significant. Thus, we concluded that aa 284–293 of RGMa binds to neogenin.

### Peptide 4 blocks RGMa-neogenin signaling *in vitro*

**Figure 3:**
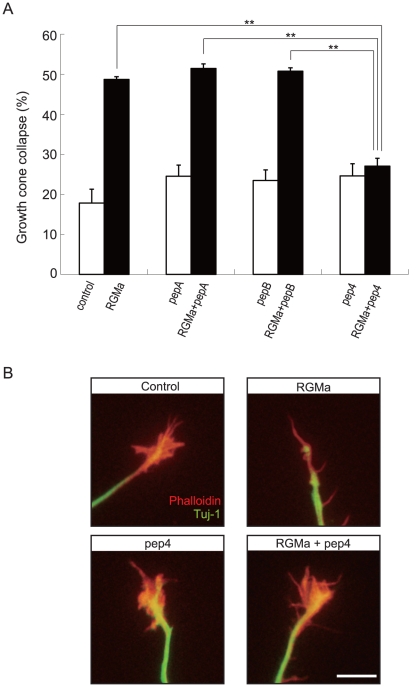
RGMa peptide 4 (aa 284–293) inhibits RGMa-induced growth cone collapse. (A) Effects of RGMa peptide 4 on RGMa-induced growth cone collapse. Cortical neurons were treated with RGMa and/or RGMa peptides for 30 min. The results were quantified from 3 independent experiments, and the percentage of collapsed growth cones is shown (**P<0.01). Pep4, peptide 4; PepA, Peptide A; PepB, peptide B. (B) Representative images of growth cones. Red: Phalloidin, Green: Tuj1. Scale bar: 10 µm.

Our results suggest that peptide 4 binding to neogenin either elicits or inhibits downstream RGMa signaling. To clarify this issue, we performed a growth cone collapse assay, since addition of RGMa to cultured cortical neurons has been shown to cause the collapse of their growth cones [13]. We cultured cortical neurons from E18 mouse for 48 h to allow sufficient neurite growth for growth cone evaluation. Recombinant RGMa was then added to the culture with or without peptide 4 (Fig. 3A and B). RGMa stimulation significantly induced growth cone collapse compared with the control (without RGMa stimulation). Addition of peptide 4 abolished the effect of RGMa, whereas addition of peptide 4 by itself did not affect growth cone behavior. We synthesized two peptides (peptide A and peptide B) as negative controls. Peptide A is a scrambled derivative of peptide 4, whereas peptide B includes aa 230–239 and is a part of the RGMa C-terminus but is not included in C-RGMa4. These peptides did not modulate RGMa-induced growth cone collapse (Fig. 3A). These results demonstrate that peptide 4 attenuates the effect of RGMa on the neurons, but does not act as an RGMa agonist.

## Discussion

Through co-immunoprecipitation studies we have shown here for the first time that the RGMa region containing aa 259–295 is sufficient for binding to neogenin. Moreover, we identified that the region containing aa 284–293 critically regulates the interaction between RGMa and neogenin. Our results also underline the physiological importance of this interaction since inhibition of this interaction by addition of RGM peptide 4 described here can attenuate the RGM-induced inhibitory effect on cortical neurons.

We demonstrate that peptide 4 attenuated the effect of RGMa on the neurons, but did not work as an RGMa agonist. Therefore, this sequence by itself may not be sufficient for the signal transduction mediated by RGMa to neogenin. We could not detect the inhibition of RGMa binding to neogenin by peptide 4 in the transfected cells by using co-immunoprecipitation (data not shown). It is possible that RGMa has multiple binding sites. However, we would like to note that the sequence we identified is functionally important. Because peptide 4 attenuated the effect of RGMa on growth cone behavior, our data demonstrate that peptide 4 sequence is required for RGMa-mediated signal transduction.

In vertebrates, 3 homologues of RGM, RGMa, RGMb, and RGMc, have been found. RGMs share common structural features, including an N-terminal signal peptide; an RGD site, which may be associated with cell attachment; a partial vWD domain; a hydrophobic domain; and a C-terminal end necessary for attaching the protein to the cell membrane via a GPI-anchor [2], [3]. The identified binding site of RGM for neogenin is not located in any of these domains; however, this binding sequence is highly conserved, at least in rat and chick RGMa, suggesting that this sequence could be physiologically important.

The RGM-neogenin interaction regulates various functions in the developing central nervous system (CNS), such as pathfinding of retinotectal projection [14], neuronal cell death [15], and neural tube closure [6]. In addition, RGM expression is induced after injury to the adult CNS [16], [17]. We have reported that inhibition of RGMa signaling by treatment with an anti-RGMa blocking antibody enhances axon regeneration and promotes functional recovery after spinal cord injury [9]. Furthermore, we recently reported that RGMa is expressed in dendritic cells, and neogenin is expressed in helper T cells [18]. RGMa enhances helper T-cell activation via dendritic cells both *in vitro* and *in vivo*. Importantly, inhibition of RGMa signals by neutralizing antibody blocked experimental autoimmune encephalomyelitis and T-cell activation in peripheral blood mononuclear cells from multiple sclerosis subjects. These findings suggest that RGMa is a promising molecular target to treat CNS injury, as well as, multiple sclerosis. Based on these observations, the peptide developed here could be used as a peptide lead for drug discovery.

Since the neogenin-binding site on RGM is located within aa 284–293, and the peptide based on this sequence inhibits RGM signaling *in vitro*, it seems likely that this peptide competitively inhibits the interaction between RGM and neogenin, thereby leading to attenuation of the effect of RGMa on neurons. The identified neogenin-binding site on RGMa is different from the antigen sequence of the anti-RGMa neutralizing antibody previously reported [9]. Supporting our findings in the present study, this neutralizing antibody does not inhibit the binding of RGMa to neogenin (unpublished data), suggesting that the region recognized by the antibody may be important for the receptor function. For example, it is possible that this region is required for interaction of neogenin with its co-receptor Unc5B [13]. Future study will determine functionally important domains in RGMa by elucidating the protein structure of RGMa.

Our findings provide important information on the function of RGMa and suggest that small molecules, peptides, and neutralizing antibodies targeting the region of RGMa identified in this study would be effective in blocking RGMa signaling *in vivo*. These reagents may be used to treat patients with spinal cord injury and multiple sclerosis.

## Materials and Methods

The Institutional Animal Care and Use Committees of Graduate School of Medicine, Osaka University approved all experimental procedures (19-081-0).

### Antibodies and reagents

The following primary antibodies were used in this study: Myc-tag (1∶1,000; Santa Cruz Biotechnology, Santa Cruz, CA) and vesicular stomatitis virus glycoprotein (VSV-G)-tag (1∶1,000; Sigma-Aldrich, St. Louis, MO). Horseradish peroxidase (HRP)-conjugated anti-mouse and anti-rabbit IgG secondary antibodies (Cell Signaling Technology, Danvers, MA, USA), and the ECL plus system (GE Healthcare Chalfont St Giles, England) were used for detection.

### Plasmid constructs

To generate a secreted form of RGMa, cDNA corresponding to human RGMa (aa 1–422), which lacks a C-terminal GPI anchor region was generated [19]. The constructs FL-RGMa, N-RGMa, C-RGMa1, C-RGMa2, C-RGMa3, C-RGMa4, and C-RGMa5 (sequence information is provided in Fig. 1C) were cloned into pSecTag2C/Hygro (Invitrogen, Carlsbad, CA, USA). All constructs were verified by DNA sequencing. VSV-G-tagged human neogenin in pcDNA 3.1 was a kind gift from Dr. Eric R Fearon [20], [21], [22].

### Cell culture and transfection

HEK293T cells (ATCC, Manassas, VA) were cultivated in Dulbecco's modified Eagle medium (DMEM) (Invitrogen) supplemented with 10% (v/v) fetal bovine serum (FBS) and penicillin/streptomycin (Invitrogen). These cells were transfected with lipofectamine 2000 (Invitrogen) according to the manufacturer's instructions.

For cortical neurons, E18 mouse embryo cortices were isolated and dissociated with 0.25% trypsin (Invitrogen) and 0.5 mg/mL DNase1 (Sigma-Aldrich, St Louis, MO, USA) for 15 min at 37°C. DMEM (Invitrogen) containing 10% FBS was added, and the cells were centrifuged at 1,000 rpm for 4 min. Neurons were plated on poly-l-lysine-coated chamber slides and maintained in DMEM/F12 medium containing B27 supplement (Invitrogen) at 37°C in an atmosphere of 5% CO_2_.

### Co-immunoprecipitation

For co-immunoprecipitation of neogenin with RGMa, HEK293T cells were transfected with VSV-G-tagged neogenin and Myc-tagged RGMa constructs and incubated for 36–48 h. After washing, the cells were lysed in 1% Triton X-100, 150 mM NaCl, 20 mM Tris/HCl (pH 7.5), complete EDTA-free protease inhibitors (Roche Diagnostics, Basel, Switzerland), and 1 mM phenylmethylsulfonyl fluoride. VSV-G antibody (2 µg) was added to the cell lysates and incubated for 4 h under rotation at 4°C followed by the addition of protein A-sepharose beads (GE Healthcare) and a second 4-h incubation under rotation at 4°C. Protein precipitates were washed several times, and were then separated by sodium dodecyl sulfate polyacrylamide gel electrophoresis and immunoblotted to detect Myc-tagged RGMa.

### Peptide synthesis

Six peptides were synthesized (SIGMA Life Science, Japan) for use in ELISA. Each peptide consisted of 10 aa encoding RGMa (covering aa 257–315, Fig. 2A) fused with an N-terminal TAT protein (YGRKKRRQRRR). RGMa peptide 4 without a TAT protein was synthesized in the same way and was used in the growth cone collapse assay. For growth cone collapse assay, three peptides were synthesized in the same way. Peptide 4 without TAT protein; peptide A, scrambled peptide of peptide 4 (QLGYFRVATR); and peptide B, aa 230–239. Peptide A and B were used as negative controls.

### ELISA

ELISAs were performed using 96-well microplates (Thermo Fisher Scientific, Waltham, MA, USA) coated with 0.1% bovine serum albumin (BSA)/phosphate-buffered saline (PBS) or synthetic RGMa peptides (5 µg/mL, 100 µL/well) for 16 h at 4°C. After washing with PBS, the wells were blocked for 1 h with 5% BSA/PBS. Recombinant mouse neogenin-Fc chimera protein (R&D Systems, Minneapolis, MN, USA) or p75-Fc chimera protein (R&D systems) as a control was then added (1 µg/mL, 100 µL/well) to the plates. One hour after incubation, the plates were washed, and anti-IgG Fc antibody was added. HRP-conjugated secondary antibodies, substrate reagent, and stop solution (R&D Systems) were used to detect protein binding. Absorbance was measured at 450 nm.

### Growth cone collapse assay

Cortical neurons were isolated from E18 mouse embryos and plated onto 3.5-cm dishes coated with poly-l-lysine (100 µg/mL, Sigma-Aldrich). Cells were cultured in an incubator gassed with 5% CO_2_ in DMEM/F12 medium containing B27 supplement (Invitrogen). After 48 h in culture, cells were treated with recombinant RGMa (2 µg/mL) and/or RGMa-peptide 4 (1 µM) for 30 min. Cells were fixed with 0.5% glutaraldehyde for 30 min at room temperature and treated with PBS containing 5% BSA and 0.1% Triton X-100. The cells were then incubated with anti-neuronal class III ß-tubulin (Tuj1, 1∶5000; Covance Laboratories, Inc., Berkeley, CA, USA) and rhodamine-phalloidin (Invitrogen) for 2 h at room temperature, and secondary anti-mouse IgG antibody conjugated with Alexa Fluor 488 (Invitrogen) for 1 h at room temperature. Neurons were analyzed using an OLYMPUS BX51 microscope. The criteria for collapsed growth cones were a total loss of filopodia and lamellipodia.

### Statistical analysis

In Fig. 2B and 3A, the results (n = 3) were analyzed using single-factor analysis of variance followed by Scheffe's multiple comparison test. *P* values of less than 0.05 were considered significant.
